# Editorial: Ketogenic metabolic therapy as a treatment for mental health disorders

**DOI:** 10.3389/fnut.2025.1606634

**Published:** 2025-04-29

**Authors:** Georgia Ede, Beth A. Zupec-Kania, Susan A. Masino

**Affiliations:** ^1^Independent Researcher, Newburyport, MA, United States; ^2^Ketogenic Therapies, LLC, Milwaukee, WI, United States; ^3^Psychology/Neuroscience, Trinity College, Hartford, CT, United States

**Keywords:** ketogenic diet, metabolic psychiatry, schizophrenia, bipolar disorder, addiction, clinical research, translational research

For more than 75 years, biological treatments for mental illness have centered primarily around pharmaceutical interventions intended to address underlying neurotransmitter system dysfunction. This medication-oriented care model was revolutionary for its time, but unfortunately, even the most effective psychotropic medications leave the majority of people with mental illness without meaningful relief (1). Furthermore, the prevalence of mental health disorders continues to rise around the world, even in wealthy countries where most people have access to state-of-the-art psychopharmacological services (2). This alarming trend strongly suggests that environmental risk factors common to communities around the globe may be contributing to widespread declines in mental wellness.

Insulin resistance, pre-diabetes, type 2 diabetes, obesity, and other metabolic disorders are becoming increasingly commonplace around the world, and are strongly associated with mental health disorders of many kinds (3). While metabolic dysfunction negatively impacts all organ systems, the brain is arguably more vulnerable than most, because it is disproportionately metabolically demanding: despite comprising only about 2% of body weight, the brain consumes about 20% of the body's energy supply (4).

The rapidly emerging field of *metabolic psychiatry* seeks to understand and address the role metabolic dysfunction plays in mental illness, generating new scientific and clinical insights that are laying the groundwork for a 21^st^ century paradigm shift in mental healthcare. The field urgently needs innovative treatment approaches that can address the metabolic disturbances commonly observed in mental health disorders (and mitigate the metabolic side effects of psychotropic medications), and mounting evidence suggests that ketogenic metabolic therapy has the potential to help meet both of these needs. Successfully used since the 1920s to treat epilepsy, ketogenic metabolic therapy has increasingly become the focus of researchers and clinicians seeking new approaches to a wide variety of other neuropsychiatric disorders as well. This Research Topic seeks to represent the depth and breadth of work being conducted in this new subspecialty, including theoretical perspectives, mechanistic research, case reports, and clinical trials.

Among the original clinical research papers is a retrospective qualitative analysis by Bellamy et al. of people's experiences with calorically unrestricted low-carbohydrate diets, noting benefits such as renewed purpose among those previously experiencing feelings of depression, as well as improvements in self-esteem, confidence, and other subjective measures important to quality of life not often formally assessed in metabolic research.

Calabrese et al. present a case series of three adults who achieved complete remission from both treatment-resistant major depressive disorder and generalized anxiety disorder after engaging in a 12–16 week lifestyle protocol centered around a ketogenic diet.

Edwards et al. presents the first pilot trial of the ketogenic diet in post-traumatic stress disorder, documenting acceptability and clinical benefits in two of the three individuals who completed the 4-week protocol, and highlighting challenges for future clinical trials.

Laurent details the case of a woman with bipolar disorder whose depression had responded only minimally to weekly ketamine treatments. Ketogenic metabolic therapy led to measurable improvements not only in depression, but also in anxiety and PTSD symptoms, as well as in measures of daily function, mental well-being, and quality of life. In a separate perspectives paper, Laurent encourages metabolic psychiatry researchers to collect and analyze both qualitative and quantitative data to present a fuller picture of the impact ketogenic metabolic therapy can have on the lives of people with mental illness.

Winje et al. report about a patient with type I diabetes who was able to stabilize blood glucose levels using ketogenic metabolic therapy, reducing fear of hypoglycemia as well as alleviating anxiety and depression symptoms.

Especially noteworthy is a paper by Longhitano et al. detailing the protocol they are implementing in a clinical trial involving 100 adults with schizophrenia and bipolar disorder, already under way in Australia. As this will be the world's first randomized controlled trial of the ketogenic diet in serious mental illness, their findings are eagerly anticipated.

Additional articles offer perspectives on the potential utility of ketogenic diets in the management of neuropsychiatric conditions beyond mood and psychotic disorders.

An intriguing review paper by Frank and Scolnick presents hopeful emerging evidence suggesting that properly formulated ketogenic diets, despite commonly being viewed as weight loss interventions, may support people in their recovery from anorexia nervosa, a condition with a high fatality rate and no approved biological treatment.

Ruskin et al. explore how ketosis positively influences the adenosine system, the dopamine system, and relevant factors such as inflammation, thereby representing a long-overlooked opportunity to support people suffering with addictive disorders. O'Hearn offers a conceptual analysis of the relationship between energy status, metabolic state, and sleep regulation which may help to explain the positive effect of ketosis on sleep quality. Stanton puts forth hypotheses on how well-formulated ketogenic and carnivore diets could stabilize certain factors associated with migraine headaches. Grabowska et al. point out, based on a review of 90 studies of the ketogenic diet conducted in rodent models, that behavioral health outcomes such as anxiety and depression have been less promising in these animal models than those observed in human case reports and clinical trials, a phenomenon also observed in epilepsy research (5).

Gertler and Blackford highlight the underexplored potential of ketogenic metabolic therapy in the management of pediatric mental and metabolic health disorders, particularly those that often coexist in children with epilepsy such as ADHD, autism spectrum disorder, and childhood obesity.

Diamond et al. challenge the widespread concern that ketogenic diets jeopardize cardiovascular health because they are high in fat and sometimes lead to elevations in LDL cholesterol—a persistent obstacle to wider acceptance of ketogenic diets by clinicians and patients alike.

The Centers for Disease Control estimate that more than 50% of Americans will be diagnosed with a mental health disorder in their lifetime (6), so the need for novel approaches to understanding, treating, and perhaps even preventing these burdensome conditions could not be more urgent. The papers curated for this Research Topic represent a diversity of efforts aimed at improving the lives of individuals with a range of neuropsychiatric conditions through innovative research into brain metabolism and the supervised incorporation of ketogenic metabolic therapy into clinical care. These works strengthen our understanding of the important relationship between metabolic health and mental health, and contribute to the growing sense that ketogenic metabolic therapy is emerging as a powerful, low-risk, lifestyle-based tool that could be integral in paving the path to a more hopeful future for mental health practitioners and the patients they serve.
